# Selection for complex traits leaves little or no classic signatures of selection

**DOI:** 10.1186/1471-2164-15-246

**Published:** 2014-03-28

**Authors:** Kathryn E Kemper, Sarah J Saxton, Sunduimijid Bolormaa, Benjamin J Hayes, Michael E Goddard

**Affiliations:** Department of Agriculture and Food Systems, University of Melbourne, Parkville, 3052 Australia; Australian Dairy Herd Improvement Scheme, 22 William Street, Melbourne, 3000 Australia; Department of Environment and Primary Industries, AgriBio, Bundoora 3086 Australia; La Trobe University, Bundoora, 3086 Australia; Dairy Futures Co-operative Research Centre, Bundoora, 3086 Australia

## Abstract

**Background:**

Selection signatures aim to identify genomic regions underlying recent adaptations in populations. However, the effects of selection in the genome are difficult to distinguish from random processes, such as genetic drift. Often associations between selection signatures and selected variants for complex traits is assumed even though this is rarely (if ever) tested. In this paper, we use 8 breeds of domestic cattle under strong artificial selection to investigate if selection signatures are co-located in genomic regions which are likely to be under selection.

**Results:**

Our approaches to identify selection signatures (haplotype heterozygosity, integrated haplotype score and *F*_*ST*_) identified strong and recent selection near many loci with mutations affecting simple traits under strong selection, such as coat colour. However, there was little evidence for a genome-wide association between strong selection signatures and regions affecting complex traits under selection, such as milk yield in dairy cattle. Even identifying selection signatures near some major loci was hindered by factors including allelic heterogeneity, selection for ancestral alleles and interactions with nearby selected loci.

**Conclusions:**

Selection signatures detect loci with large effects under strong selection. However, the methodology is often assumed to also detect loci affecting complex traits where the selection pressure at an individual locus is weak. We present empirical evidence to suggests little discernible ‘selection signature’ for complex traits in the genome of dairy cattle despite very strong and recent artificial selection.

**Electronic supplementary material:**

The online version of this article (doi:10.1186/1471-2164-15-246) contains supplementary material, which is available to authorized users.

## Background

Evolutionary change in a population, in response to a change in environment, consists of an increase in the frequency of favourable mutations. If the mutation was recent and the selection is strong, all alleles on the same chromosome segment as the mutant allele will increase in frequency by hitchhiking, generating a characteristic selection sweep or selection signature [1]. On the other hand, if selection at individual loci is weak or if the mutation is old, and therefore part of the standing variation when selection commences, little evidence of the selection may be left in the genome e.g. [2]. Many statistics have been proposed to detect signatures of selection but they all suffer from a severe problem – the distribution of the statistic under the null hypothesis of no selection is usually unknown. This is because the distribution depends on the demography of the population, including changes in effective population size and migration, which are difficult to define. Consequently, no formal test that a statistic comes from the null distribution is possible. Generally, the most extreme values of the statistic are simply assumed to be due to selection and there have been many papers claiming to find evidence for signatures of selection. The evidence for selection sweeps at a small number of loci, such as for lactase persistence in humans [3] and skin wrinkling in Shar-Pei dogs [4], is well documented and convincing, but in other cases it is hard to evaluate the strength of evidence. Certainly the evidence and persuasiveness of authors advocating adaptation via standing polymorphisms is increasing [5–7] and the influential paradigm of ‘hard sweep’ selection signatures is beginning to lose favour as the primary mechanism of adaptation [1].

In this study we have taken a different approach – we study sites in the genome at which we know selection has occurred to see if a signature of selection has been left behind. By studying a variety of selected loci, we are able to describe when a selection signature is generated and when it is not. Domestic cattle have been under quite strong, recent and well documented selection for several traits and hence their genomes should contain evidence of this selection. We use 8 domestic *Bos taurus* cattle breeds and three types of loci which have been under selection: type 1 loci are genes that are part of the definition of a breed, such as absence of horns and coat colour; type 2 loci have a large effect on quantitative traits, such as stature and milk yield, and type 3 loci are quantitative trait loci (QTL) for milk production traits in dairy cattle. We consider two statistics that indicate selection signatures within a breed and *F*_*ST*_ (which indicates a difference between breeds in a segment of the genome that could be caused by different selection histories between the breeds). Our results show clear signatures of selection when intense selection has been applied to a single locus because it causes a trait defining the breed such as coat colour. However, we find weak evidence for selection signatures at regions of the genome associated with complex traits under selection. This paper calls into question the reliability of selection signatures to identify mutations affecting complex traits under selection and provides empirical evidence for the ability to generate substantial genetic change between populations in complex traits without clear evidence for classic selection signatures.

## Results

### Measures of selection

The dataset consists of 23,641 domestic cattle with > 610,123 (real or imputed) genome-wide autosomal SNP from 8 *B. taurus* breeds. Breeds were of European origin and have had previous, recent selection for milk (Holstein, Jersey) or meat (Angus, Charolais, Hereford, Limousin, Murray Grey, Shorthorn) production. There were between of 61 (Limousin) and 13,501 (Holstein) animals genotyped per breed.

Three statistics were calculated to test for evidence of selection: a modified version of Depaulis-Veuille’s H-test (referred to as haplotype homozygosity, *HAPH*) [8], the integrated haplotype score (*|iHS|*) [4], and Wright’s measure of population differentiation (*F*_*ST*_). The measure of haplotype homozygosity (*HAPH*) measures selection within breed and is defined as the variance of haplotypes frequencies at a particular position in the genome, i.e.  where *p*_*i*_ is the (within breed) frequency of the *i*^th^ haplotype and *N* is the total number of haplotypes at the position. The haplotypes consist of 30 or 31 consecutive SNPs. This statistic is high if one or more haplotypes are at high frequency while most haplotypes exist at low frequency. Similarly, *|iHS|* identifies within breed selection and SNP where one allele is found on one or few long haplotypes whereas the other allele is associated with many haplotypes. Both *HAPH* and *|iHS|* are efficient for identification of sweeps which have not yet reached fixation, an essential feature for an association with type 3 loci (i.e. genomic regions with segregating mutations for complex traits under selection). In contrast, the *F*_*ST*_ measurement is most efficient when there are large allele frequency differences between pairs of breeds. Selection is indicated by high values of *F*_*ST*_ near the selected mutations because, for example, a population in which selection has taken place is expected to differ from other populations (that have not undergone the same selection) in the allele frequency for markers near the mutation.

The 3 measures of selection were calculated in 250 kb windows across the genome, where the value for each window was the mean *HAPH*, the maximum observed *|iHS|* or the average between breed *F*_*ST*_. To correct for average differences within and between breeds for *HAPH* and *F*_*ST*_, the values are standardised by dividing the window value by the mean value for all windows. Consequently the standardised estimates of selection have a mean of 1. *|iHS|* was calculated following [9], and is thus standardised such that *|iHS|* can be interpreted as standard deviations from the mean. The estimates (per window) of *HAPH, |iHS|* and breed comparisons for *F*_*ST*_ are given in Additional file 1 (where Additional file 2 provides definitions of the columns for Additional file 1). We examined the 5% of the genome with the strongest evidence for selection.

Breed-defining loci (type 1) and large effect QTL (type 2) were identified from the literature and the Online Inheritance in Animals database [10]. For type 3 loci, we used the Holstein and Jersey breeds to identify QTL regions in the genome for milk production traits using the ‘genomic selection’ methodology [11]. These two breeds have been under strong selection for milk production for at least the last 100 years [12] and especially since the 1970’s (Additional file 3: Figure S1-S3). In genomic selection, the prediction of genetic merit is a linear regression in which each SNP genotype is multiplied by the estimated effect of a SNP and summed to yield an estimated breeding value (EBV) for the animal. In our case, we want to attach variation in the trait to each chromosome segment. Thus we estimated the effect of each SNP using the genomic selection methodology and then calculated the variance across animals for a local 250 kb EBV e.g. [13]. The 5% of windows with the highest variance were considered to have QTL and defined as type 3 loci.

### Breed-defining loci often showed selection signatures

There were 5 loci that control phenotypes which are characteristics of the breed. These loci are polled (i.e. absence of horns) and 4 loci (*MC1-R*, *PMEL*, *KIT* and *KITLG*) that determine coat colour (Table 1). Most of these loci (including *POLLED*, *MC1-R*, *KIT* and *PMEL*) have previously been reported as under selection e.g. [14–18] and we find evidence for all loci of within breed selection using *HAPH* (Table 2).

**Table 1:** **Description of (type 1) loci with a large effects on breed-defining traits, such as coat colour, in in domestic cattle and likely to be segregating in our populations**

Locus	Location	Description
*POLLED*	BTA11.71 Mbp	Determines the presence and absence of horns. Two identified alleles: *P*_*C*_ (Celtic-origin) a 212 bp insertion-deletion at 1.706 Mbp; and *P*_*F*_ (Holstein Friesian-origin) which segregates as a 260 kb haplotype (from 1.649 – 1.989 Mbp) in Holstein and Jersey [18, 19]. No known associated gene. Most domestic cattle are horned but Angus and Murray Grey breeds are exclusively polled and the *POLLED* locus segregates in other breeds.
*MC1-R*	BTA18 14.75 Mbp	The main determinant of coat colour in cattle [20]. Two identified alleles: *E* ^*D*^ (p.L99P) which produces a black coat; and *e* (inducing a premature stop codon) which is recessive produces a red coat when homozygous [21].
*PMEL*	BTA5 57.67 Mbp	Coat colour dilution mutation (c.64G > A) identified in Charolais [22]. Different *PMEL* mutations segregate in Highland and Charolais cattle [23].
*KIT*	BTA6 71.85 Mbp	Locus associated with piebald colour in Hereford [24] and degree of white-spotting in Holstein [25]. No known causative mutations but the different coat colour patterns in these breeds, suggests different *KIT* mutations.
*KITLG*	BTA5 18.34 Mbp	A SNP mutation (p.A193D) identified in Shorthorn and Belgian Blue as causative for the roan phenotype [26]. *KITLG* is also associated with pigmentation surrounding the eyes in Fleckvieh cattle [27].

**Table 2:** **Evidence for within and between breed selection at breed-defining (type 1) loci**

Locus	Evidence for selection^*^		
	Within breed	Differentiation between breeds^3^	
*POLLED*	Angus^1,2^	1. Holstein with Angus, Murray Grey and Limousin	Figure 1
	Charolais^1,2^		
	Holstein^1,2^		
	Limousin^1,2^		
	Hereford^1^		
	Shorthorn^1^		
*MC1-R*	Limousin^1,2^	1. Breeds with black (*E* ^*D*^) allele (Holstein, Angus, Murray Grey) with breeds with recessive red (e) allele (Charolais, Limousin, Shorthorn, Hereford)	Additional file 3: Figure S4
	Charolais^1^		
	Angus^1^	2. Jersey (E^+^ allele) with all other breeds, except Hereford	
	Holstein^1^		
	Murray Grey^1^		
*PMEL*	Charolais^1,2^	1. Charolais with all other breeds	Additional file 3: Figure S5
	Angus^2^	2. Murray Grey with all breeds, excluding Jersey	
	Murray Grey^1^	3. Shorthorn and Jersey	
*KIT*	Hereford^1,2^	1. Hereford with all other breeds.	Additional file 3: Figure S6
	Holstein^1^	2. Holstein with all breeds, except Jersey	
		3. Shorthorn with all breeds, except Jersey	
		4. Jersey with Angus, Charolais and Limousin	
*KITLG*	Hereford^1^	1. Hereford will all other breeds, except Murray Grey	Additional file 3: Figure S7
		2. Murray Grey and Charolais with each other, and with Holstein, Angus and Limousin	
		3. Shorthorn with Augus	

^*^windows encompassing loci and identified in the top 5% of within or between breed measures of selection. Measures of selection were ^1^haplotype homozygosity (*HAPH*), ^2^integrated haplotype score (*|iHS|*) and ^3^ *F*_*ST*_.

**Figure 1:**
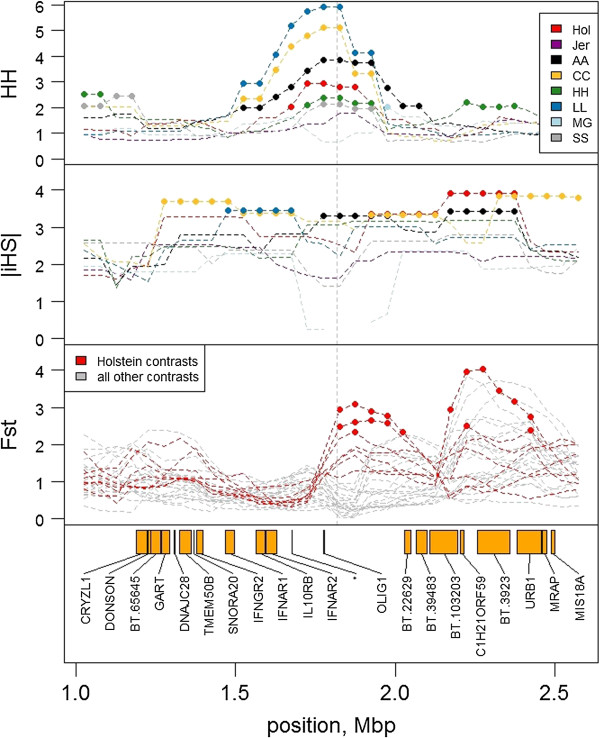
**Haplotype homozygosity (** ***HAPH*** **), the integrated haplotype score (** ***|iHS|*** **) and** ***F***_***ST***_ **near the** ***POLLED*** **locus.** Breeds are Holstein (Hol, red), Jersey (Jer, purple), Angus (AA, black), Charolais (CC, yellow), Hereford (HH, green), Limousin (LL, blue), Murray Grey (MG, light blue) and Shorthorn (SS, grey). Points indicate windows with extreme (top 5%) values for *HAPH*, *|iHS|* or *F*_*ST*_. *F*_*ST*_ of each breed with Holstein are highlighted in red (bottom panel). Trait units are multiples of an average window (HH, FST) or absolute standard deviations from the mean (|iHS|).

There is evidence for more than one selected mutation at each of the type 1 loci. This evidence includes selection within 2 or more breeds but large *F*_*ST*_ between these selected breeds as well as between each selected breed and the breeds not selected at this gene. For example, near *POLLED* we found within breed selection signatures (i.e. top 5% of window *HAPH* values) for Limousin, Charolais, Angus, Holstein, Hereford, Murray Grey and Shorthorn and across-breed differentiation (i.e. top 5% of *F*_*ST*_ values) for Holstein with Angus, Murray Grey and Limousin (Figure 1). This is consistent with the 2 different reported mutations for *POLLED*[18, 19], where the *P*_*C*_ allele segregates in Angus, Charolais, Limousin and Hereford and the *P*_*F*_ allele segregates in Holstein. Selection signatures near *POLLED* in Western European cattle are also thought to pre-date *P*_c_ mutation [18], indicating the possibility of further (as yet undescribed) alleles. We also propose allelic heterogeneity for *PMEL* in Charolais and Murray Grey cattle, where both breeds show strong within breed selection using *HAPH* but a large value of *F*_*ST*_ between them (Additional file 3: Figure S5). Different *PMEL* mutations are known to segregate in Charolais and Scottish Highland cattle [23], and here it appears the Charolais mutation is also different to a *PMEL* mutation in Murray Grey.

The observed frequency of the selected haplotype played an important role in determining the ability of the three test statistic to indicate selection. At *POLLED*, for example, neither *HAPH* nor *|iHS|* indicated evidence of within breed selection in Murray Grey despite all animals of this breed being polled. This is because this region is homozygous in Murray Grey and neither of these statistics indicates selection in homozygous regions, being either undefined (*|iHS|*) or with values close to zero (*HAPH*). Further at *PMEL,* long selected haplotypes were indicated by *HAPH* and *F*_*ST*_ in Murray Grey but there was no *|iHS|* selection signature near the locus. The results show that *F*_*ST*_ is most efficient when the region is near fixation (homozygous) in alternate breeds, *|iHS|* is most efficient for intermediate frequency (or segregating) variants [9] and *HAPH* is midway between the two measures.

The mode of action and favoured phenotype also determined if loci indicated selection. In Shorthorn, for example, there was no within breed selection signature near *KITLG* despite a roan coat (where white hairs are intermingled with coloured hairs) being a characteristic of this breed [26]. This can be explained by balancing selection, where heterozygotes express the roan phenotype and homozygotes have either a solid coloured or white coat, which would not be efficiently detected by any method. There was also evidence for a within breed selection near *KITLG* in Hereford. Herefords do not have a roan phenotype and, considering results in Fleckvieh cattle [27], this may indicate that a *KITLG* mutation contributes to the characteristic white spotting pattern seen in Hereford and Fleckvieh.

### Selection at known loci affecting quantitative traits

There were 5 type 2 loci chosen which had large effects mutations on stature (*PLAG1*), milk production (*DGAT1, GHR, ABCG2*) and muscle mass (*MSTN*) (Table 3). These loci were examined for the presence of selection signatures and, for *DGAT1*, *GHR* and *ABCG2*, to confirm their effect on milk production (Table 4). Selection signatures indicating selection in dairy, as compared to beef, breeds have previously been reported for *GHR* and *ABCG2*[14, 28], while other loci (*PLAG1, DGAT1* and *MSTN*) have previous reported selection signatures e.g. [17, 29, 30].

**Table 3:** **Description of (type 2) loci with large effects on complex traits under selection in domestic cattle, such as milk and meat yield, and likely to be segregating in our populations**

Locus	Location	Description
*PLAG1*	BTA14 25.00 Mbp	Region affecting many traits, including stature [31] and fertility [29]. Originally identified in Jersey-Holstein cross, Jersey are thought to be near fixation for the ancestral allele while Holstein and other breeds are near fixation for the alternate allele [29, 32].
*DGAT1*	BTA14 1.80 Mbp	Dinucleotide substitution causing a lysine to alanine substitution (p.K232A) [33], where the mutant A allele decreases fat yield, and increases protein yield and milk volume [34, 35]. The mutant *DGAT* ^*A*^ allele is at high frequency or fixed in Hereford, Angus and Charolais; and at lower frequencies in Holstein and Jersey [35].
*GHR*	BTA20 32.05 Mbp	A SNP mutation causing a missense phenylalanine to tyrosine substitution (p.F279Y). Effects on milk volume and composition [36].
*ABCG2*	BTA6 37.97 Mbp	A SNP mutation causes a missense tyrosine to serine (p.Y581S) mutation which increases milk yield and decreases milk solids [37]. Identified in Israeli Holsteins where the frequency of the *ABCG2* ^*C*^ allele had increased in response to selection for milk yield and then decreased when selection changed to focus on increased milk solids [37]. The *ABCG2* ^*C*^ allele is at low frequencies (< 10%) in US and German Holsteins, Angus, British Frisian, Charolais and Hereford [38].
*MSTN*	BTA2 6.22 Mbp	A negative regulator of muscle development, multiple mutations have been described that cause ‘double muscling’ or extreme muscular hypertrophy [32, 39, 40]. In Limousin, a mutation associated with a mild increase in muscling, F94L, has been identified [41].

**Table 4:** **Evidence for selection and quantitative trait loci (QTL) at major loci affecting complex traits (type 2 loci)**

Locus	Evidence for selection^*^		Evidence for dairy QTL^**^	
	Within breed	Differentiation between breeds^3^		
*PLAG1*	Holstein^1,2^	1. Jersey with all other breeds	NA.	Figure 2
	Charolais^1,2^	2. Limousin with all breeds, except Hereford		
	Shorthorn^1,2^	3. Hereford with all breeds, except Limousin and Angus		
	Angus^1^	4. Murray Grey with all breeds, except Shorthorn and Holstein		
	Limousin^1^			
	Hereford^1^			
	Murray Grey^1^			
*DGAT1*	Limousin^1,2^	1. Holstein or Jersey with Charolais, Limousin, Hereford and Shorthorn	Holstein and Jersey: Milk yield, fat yield, protein yield, FPC and PPC.	Additional file 3: Figure S8
	Angus^1^	2. Murray Grey with Hereford		
	Charolais^1^			
	Hereford^1^			
	Murray Grey^1^			
	Shorthorn^1^			
*GHR*	Holstein^1,2^	1. Holstein with Jersey, Charolais & Limousin	Holstein: Milk yield, fat yield, protein yield, FPC and PPC.	Additional file 3: Figure S9
	Jersey^2^	2. Angus with Jersey, Charolais & Murray Grey	Jersey: Milk yield, FPC and PPC.	
		3. Jersey with Holstein, Angus & Shorthorn		
*ABCG2* ^***^	Jersey^1,2^	1. All contrasts between Jersey, Hereford and Charolais	Holstein: Fat yield, protein yield and PPC.	Additional file 3: Figure S10
	Charolais^1,2^		Jersey: Stature.	
	Limousin^2^			
*MSTN*	Limousin^1^	1. Limousin with all other breeds	NA.	Additional file 3: Figure S11

^*^windows encompassing loci and identified in the top 5% of within or between breed measures of selection. Measures of selection were ^1^haplotype homozygosity (*HAPH*), ^2^integrated haplotype score (*|iHS|*) and ^3^ *F*_*ST*_.

^**^traits in Holstein and Jersey dairy cattle are milk yield (litres per lactation), fat yield (kg per lactation), protein yield (kg per lactation), FPC (fat percentage in milk), PPC (protein percentage in milk) and stature.

^***^within breed selection for Charolais at *ABCG2* is probably for *NCAPG* (at 38.78 Mbp).

NA = not applicable, QTL not expected to segregate in Holstein and Jersey cattle.

**Figure 2:**
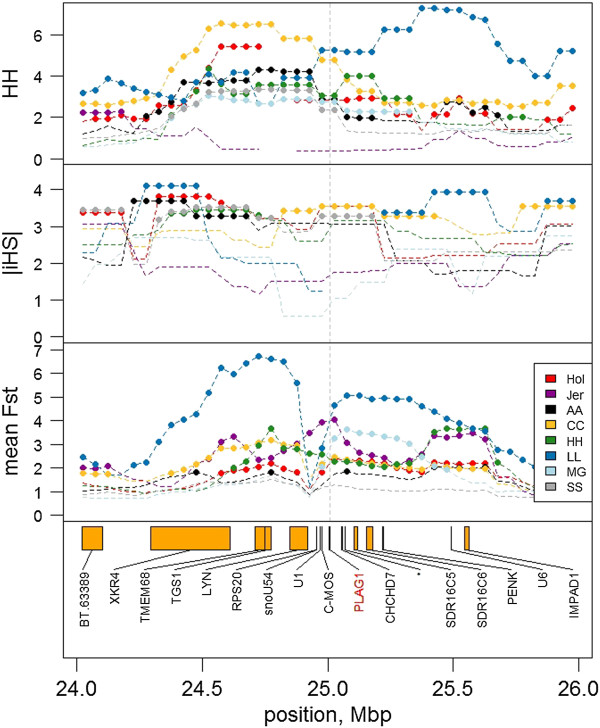
**Haplotype homozygosity (** ***HAPH*** **), the integrated haplotype score (** ***|iHS|*** **) and mean** ***F***_***ST***_ **near** ***PLAG1*** **.** Breeds are Holstein (Hol, red), Jersey (Jer, purple), Angus (AA, black), Charolais (CC, yellow), Limousin (LL, blue), Murray Grey (MG, light blue) and Shorthorn (SS, grey). Points indicate windows with extreme (top 5%) values for *HAPH*, *|iHS|* or *F*_*ST*_. For simplicity, *F*_*ST*_ is presented as the mean for each breed with all other breeds. Trait units are multiples of an average window (*HH, F*_*ST*_) or absolute standard deviations from the mean (*|iHS|*).

We find evidence for selection signatures near all type 2 loci, but the evidence had greater ambiguity than for the breed-defining (type 1) loci in most cases. The notable exception was at *MSTN*, where there was clear evidence of recent and strong selection in the Limousin breed (Table 4, Additional file 3: Figure S11). The other loci showed more ambiguous patterns of selection. In the case of *ABCG2* and *GHR*, this was likely to be because selection signatures were affected by several mutations in the region. For example, near *ABCG2* there is a strong selection signature in Charolais, probably due to selection at the *LCORL* or *NCAPG* locus [17, 42], and there appears to be several QTL for milk production traits in BTA20 near *GHR*[43]. In other cases, such as *PLAG1*, a more complex pattern of selection arises (Figure 2). For instance, Limousin differ from other breeds for most windows in the region except a window centred near *LYN* and incorporating *PLAG1*. Limousin seem to have the same haplotype as other breeds in the immediate *LYN-PLAG1* region but differentiate in the surrounding region. This could be explained if the mutation was introduced into Limousin from another breed and one hybrid haplotype became the common ancestor for most Limousin haplotypes in the region.

Aligning selection signatures and QTL in dairy cattle was also not always straight forward. Sometimes this was because alleles did not segregate within the dairy breeds and sometimes because recent selection was for the ancestral (rather than the derived) allele. For example, there was no stature QTL for Holstein or Jersey near *PLAG1* because Jerseys have a high frequency of the ancestral allele and Holstein have a high frequency of the (proposed) mutant allele [31]. Further, our QTL results confirm the segregation of the *DGAT1* mutation in both dairy breeds (Jersey and Holstein) but *DGAT1* showed within breed selection signatures only in the beef breeds. It is possible that selection some time ago was for the mutant allele (in both dairy and beef cattle) because it increased milk volume but more recent selection in Jersey and Holstein has been for the ancestral allele because it increases milk fat. Thus the recent selection in dairy breeds is not detected within either Jerseys or Holsteins because selection has been for the ancestral allele which is likely to be carried on a variety of haplotype backgrounds and so is unlikely to show a discernible selection signature.

### Has selection for milk production left selection signatures in dairy cattle?

Type 3 loci are regions of the genome which show genetic variation in Holstein and Jersey cattle for 7 different production traits (fat, milk and protein yield; stature; fertility; and percentage of fat and protein in milk). Most of these traits have been under strong recent selection (Additional file 3: Figure S1-S3). We used a chi-squared test to investigate if there was greater overlap, than expected by chance, between the windows identified as containing QTL (i.e. type 3 loci, top 5% of windows with the highest variance) and windows identified with selection signatures (i.e. top 5% of *HAPH*, *|iHS|* or *F*_*ST*_ values). The within breed measures of selection (*HAPH*, *|iHS|*) assess haplotype frequencies and should be efficient at detecting on-going recent selection while, in contrast, high *F*_*ST*_ between dairy by beef breeds will identify areas of the genome where there is differentiation between dairy and beef breeds, but not within either group.

Overall, there was a relatively weak association between QTL and selection signatures (Table 5). There was evidence for an association between *|iHS|* and QTL for protein yield in Holstein and between *|iHS|* and QTL for stature in Jersey (P < 0.05, Bonferroni corrected). There were 1.6 and 1.8 times the number of windows with QTL and high *|iHS|* than expected by chance. There was no association between selection as measured by *HAPH* or dairy-beef *F*_*ST*_ and any traits. This is despite the strong correlation between *|iHS|* and *HAPH*, where 2.8 and 5 times more windows were identified in the top 5% of *HAPH* and *|iHS|* than expected by chance (for Holstein and Jersey respectively). Increasing the proportion of the genome considered to contain QTL and showing selection signatures did lead to a weak association between selection signatures and QTL. For example, the number of windows in top 20% for *|iHS|* and QTL variance was about 1.15 times the number expected by chance for all traits, with the exception of fat and protein percentage in milk for Jersey. This weak association was nevertheless significant (P < 0.05, Bonferroni corrected). Thus our data supports weak selection across many loci for most production traits.

**Table 5:** **Association between measures of selection and genome-wide quantitative trait loci (i.e. type 3 loci) in Holstein and Jersey cattle**

		FAT	MILK	PROT	STAT	FERT	FPC	PPC
(a) QTL Holstein	*HAPH* Holstein	31.4	32.8	35.6	34.0	31.0	30.6	30.8
(b) QTL Jersey	*HAPH* Jersey	21.4	25.2	29.2	22.6	20.4	16.6	19.8
(c) QTL Holstein	*|iHS|* Holstein	40.0	39.0	47.0^*^	40.2	39.6	36.0	34.6
(d) QTL Jersey	*|iHS|* Jersey	31.8	36.4	35.0	43.0^*^	34.2	28.6	27.6
(e) QTL Holstein or Jersey	*F*_*ST*_ Dairy vs. Beef	55.2	47.0	48.0	42.6	44.0	44.0	45.8
(f) QTL Holstein	QTL Jersey	46.0^*^	47.6^*^	47.6^*^	51.6^*^	34.2	50.4^*^	55.2^*^

^*^Chi-squared test P < 0.05, Bonforroni corrected P-value.

Values are the average number of windows showing both selection and type 3 loci for production traits in either Holstein or Jersey cattle (a-e) across 5 sets of 250 kb windows. Also shown is the number of overlapping windows with type 3 loci in both Holstein and Jersey (f). There are approximately 32 (a-d, f) and 46 (e) windows expected by chance. Additional file 3: Tables S1-S3 contain the full chi-squared tests.

Evidence of selection was indicated by extreme (top 5%) values for haplotype homozygosity (*HAPH*), the integrated haplotype score (*|iHS|*) and Wright’s measure of population differentiation (*F*_*ST*_).

Traits analysed for type 3 loci are: fat yield (FAT, kg per lactation), milk yield (MILK, litres per lactation), protein yield (PROT, kg per lactation), stature (STAT), fertility (FERT, calving interval), FPC (fat percentage in milk) and PPC (protein percentage in milk).

Windows with high *F*_*ST*_ values between beef and dairy breeds were not enriched for QTL affecting production traits (Table 5) even when the proportion of the genome considered was increased to 20%. Thus despite many generations of selection for increased milk production in dairy cattle, we do not find big differences in allele frequency between beef and dairy breeds near QTL for milk production. This may indicate that genetic drift between beef and dairy breeds is greater than the effects of selection. Our finding are in contrast to other studies [28], which used fewer SNP and fewer breeds than in the current analysis. However, windows containing QTL in Holstein were significantly over-represented (by 1.8 - 2.1 times) in the windows with QTL for the same trait in Jersey (Bonferroni corrected; P < 0.05), for all traits except fertility. Thus at least some QTL appear to segregate in both breeds. If the same alleles segregate in both breeds, this implies that either the polymorphisms existed since before the breeds diverged or it may be the result of admixture among our dairy cattle populations. Given that some QTL segregate across breeds, it is perhaps surprising that selection has not caused both dairy breeds to differ from the beef breeds as measured by *F*_*ST*_.

### Novel regions with strong selection sweeps in the genome

It is possible that selection has operated for traits other than those reported in Table 5 so we considered the overall prevalence of strong selection signatures in the genomes for the 8 cattle breeds. Based on long regions of high *HAPH*, there were a total of 190 regions which contained windows from the top 5% of within breed selected windows and were greater than 2 Mbp in length (Additional file 3: Figure S12) and 25 cases where sweeps were > 5 Mbp (Table 6).

**Table 6:** **Genomic regions with evidence of recent selection using haplotype homozygosity**

Breed	BTA	Sweep location & size (Mbp)			Type 1 & 2 loci
		Beginning	End	Length	
Limousin	2	0	13.85	13.85	*MSTN*
Hereford	2	68.85	74.95	6.1	
Jersey	3	38.15	47.8	9.65	
Jersey	3	50.95	57.7	6.75	
Shorthorn	3	69.75	88.4	18.65	
Angus	3	89.6	94.65	5.05	
Shorthorn	4	67.15	73	5.85	
Murray Grey	5	40.65	61.8	21.15	*PMEL*
Charolais	5	52.8	64.75	11.95	*PMEL*
Hereford	6	67.85	79.35	11.5	*KIT*
Jersey	7	36.3	48.45	12.15	
Angus	7	42.3	47.75	5.45	
Shorthorn	11	34.1	40.65	6.55	
Shorthorn	13	57.45	66.45	9	
Charolais	14	19.75	29.55	9.8	*PLAG1*
Angus	16	38.5	47.75	9.25	
Shorthorn	16	39.65	48.85	9.2	
Holstein	16	40.1	47.05	6.95	
Charolais	16	41.45	46.9	5.45	
Jersey	20	1.5	7.1	5.6	
Jersey	20	22.8	29	6.2	
Holstein	20	29.85	34.9	5.05	*GHR*
Murray Grey	22	33.2	39.45	6.25	
Murray Grey	24	22.35	29.35	7	
Holstein	26	17.6	24.3	6.7	

Six of the 25 long regions of high *HAPH* could be ascribed to the type 1 and type 2 loci. The strong selection sweep on BTA13 in Shorthorn contains the agouti (*ASIP*) locus (Table 6), which is known to affect coat colour in several species [20]. However, phenotypic expression of *ASIP* requires an agouti-susceptible allele at *MC1-R*, such as the wild-type *E*^*+*^ allele found in Jerseys [44]. Thus most of our other breeds will not show a coat colour phenotype from *ASIP* mutations. There seems to be a selected mutation specific to British breeds (i.e. Shorthorn, Angus, Murray Grey and Hereford; Additional file 3: Figure S13) and, although *ASIP* mutations are unlikely to affect coat colour in these cattle, the locus may have affected coat colour in ancestors without the *MC1-R* mutation or the mutation may affected other traits such as fatness and homeostasis [45].

Other strong selection sweeps for several breeds were located on BTA 16 (41 – 47 Mbp) and BTA 7 (42 – 47 Mbp) (Table 6). However, unlike the *ASIP* region, *F*_*ST*_ in these two regions did not indicate clear differentiation patterns between the breeds and breeds within the selected group frequently differed from each other. The selected region on BTA7 was particularly gene dense and includes, among others, 23 olfactory receptor loci. Interestingly, this region was also identified in an independent study of Fleckvieh cattle [46]. The large sweep identified in Shorthorn on BTA3 (69.75 – 88.4 Mbp) contains *LEPR* (leptin receptor, 80.1 Mbp) which has been reported to be associated with multiple growth and fatness traits in beef cattle [47]. The longest identified selected region in Holstein, where we had the largest number of genotyped animals (n = 13,501), was on BTA26. In a region also supported by a high *|iHS|* value, a promising candidate is *FGF8* (fibroblast growth factor 8 (androgen-induced)) (Additional file 3: Figure S14). There is functional evidence for the involvement of *FGF8* in lactation, as it has been found to be highly expressed in lactating (human) breast tissue and milk [48]. The selection signature on BTA3 was also identified by Stella *et al.*[15]. The region contains *SLC35A3* (solute carrier family 35 (UDP-N-acetylglucosamine (UDP-GlcNAc) transporter), member A3; at 43.4 Mbp) which is the gene at which a recessive lethal mutation causes complex vertebral malformations (CVM) in Holstein cattle [49]. A lethal recessive mutation would not cause the type of selection signature detected here but selection at a nearby linked locus could explain why the mutation in *SLC35A3* has drifted to high frequency.

Some of the long selection sweeps reported in Table 6 could be the result of random processes, such as genetic drift or demographic changes, rather than selection. However, we find that strong selection (or ‘hard’) sweeps are relatively rare in our 8 breeds of cattle. This is despite strong, recent selection for numerous traits and particularly for milk production traits in our dairy breeds. Thus one can conclude the substantial genetic improvement in milk yield in dairy cattle has not generated many clear signatures of selection.

## Discussion

We searched for selection signatures at locations in the genome which were likely to be under selection using dense SNP genotypes in the genomes of 8 domestic *B. taurus* cattle breeds. The evidence is consistent with one or more mutant alleles having been selected to high frequency in some of the eight breeds for some of loci we investigated. Consistent with a ‘hard sweep’ model of selection, the breeds carrying the mutant allele show a common long haplotype (indicated by high values of *HAPH*) and a large genetic distance (*F*_*ST*_) from the breeds carrying the ancestral allele or a different mutant allele in the region. We clearly observed this type of selection pattern at *PMEL* and *MSTN*. However, selection signatures at loci with a large effect on complex traits under selection (type 2 loci) were weaker, and almost absent for most QTL for traits under selection (type 3 loci). How can these results be explained?

A classic ‘hard sweep’ is expected when the environment changes such that a mutation that would previously been detrimental becomes favourable. Typically there is a lag and then the frequency of the favoured allele increases slowly until it reaches a modest frequency after which it is swept quickly to fixation. This is the pattern seen, for instance, in insecticide resistance [50]. Our data on *POLLED*, *MC1-R*, *KIT*, *KITLG*, *PMEL*, *PLAG1* and *MSTN* are consistent with this explanation although here the changed ‘environment’ is one in which cattle owners control which animals will be allowed to breed. The selected mutations were probably deleterious in the wild and this natural selection may still operate in domestic cattle along with the artificial selection applied by cattle owners. Therefore to drive a mutation rapidly to high frequency, artificial selection must be strong and natural selection weak. This combination is likely for some coat colour mutations – if a breed is defined to be red, then selection for a red mutation will be very strong while natural selection against the mutation may be weak, particularly if natural selection was related to environmental factors that have been reduced through the process of domestication (i.e. camouflaged from predators).

On the other hand, mutations with a large effect on growth, reproduction or milk production are likely to have detrimental side effects even under domestication. Pleiotropy is commonly observed for large-effect mutations, such as *PLAG1* affecting fertility and stature [29] or *DGAT1* affecting both milk volume and solids (fat and protein) [33], and it is unlikely that the overall effect of a particular mutation would always be favourable. Consequently, few mutations affecting these types of traits will be driven rapidly to high frequency and leave a clear selection signature. Occasionally large-effect mutations with small or inconspicuous pleiotropic effects are observed as under strong selection. We observed strong selection in Limousin at *MSTN* and there is strong, recent selection near the *PLAG1* region in Brahman cattle despite its negative effects on fertility [29].

Thus the results for type 1, 2 and 3 loci are best reconciled by considering the selection on each locus. Selection for simple (monogenic) traits applies strong selection pressure to a mutation and the results are consistent with a ‘hard sweep’ model of selection. However, complex traits in our data were not associated with classic selection signatures and ‘hard sweeps’ are relatively rare despite the recent selection for milk traits in our dairy cattle. This suggests the selection response is caused by weak selection at many sites across the genome, probably for previously segregating variants. Weak selection is expected since each QTL has a small effect the on phenotype e.g. [51, 52]. Since there are many loci, each with small effect, selection will not change the allele frequency rapidly and there will be little evidence of a selection sweep. Small changes to allele frequencies at many loci can combine to make large changes to a phenotype, consistent with the large selection response observed for the complex traits in our data. The ability to detect selection sweeps would be further hampered if selection was conducted on genetic variants already segregating in the population. Innan & Kim [53], for example, find the initial frequency of the selected alleles to be one of the primary determinants for the ability to detect a selection event using classic selection signatures.

The explanation of weak selection on old genetic variation for complex traits, although speculative, is supported by other evidence. One key and consistent observation in support of selection on standing variants is the rapid and immediate response to selection observed for most (if not all) heritable characters in domestic and experimental populations [54]. This supports frequency changes to mutations already segregating in the population because, given the rapid response, there is insufficient time for accumulation of new favourable mutations. The selection response does not usually show an acceleration, as seen with insecticide resistance, but is approximately linear and can be predicted from estimates of the genetic variance prior to selection. Nor does the selection response diminish and reach a plateau e.g. [55], except in small populations, indicating that few genes of large effect have reached fixation. Historically, debate on the mutations underlying the response to selection was divided by strong selection at a few loci or relatively weak selection at many loci. However in Holstein, for example, there has been large increases in milk production with very few ‘hard sweeps’ observed in the genome and few observations of large-effect QTL.

Although we show that most selection for complex traits does not leave a classic signature of selection, we do not imply that selection does not change the allele frequency at sites causing variation in complex traits. Turchin *et al.*[56] show that mutations affecting human height have been subject to selection because, at many loci, the alleles for increased height have higher frequency in northern than in southern Europe. However, Turchin *et al.* present no evidence that a selection signature could be discerned if the sites associated with variation in height were not already known. In human height and in cattle milk yield, selection has no doubt changed allele frequencies at causal loci but not enough to leave a selection signature that is recognisable in the absence of prior knowledge of loci associated with height or milk yield or indeed most complex traits. An implication of this conclusion is that searching for classic selection signatures is not a powerful method to map genes for complex traits even if the traits have been under selection.

Identification of genomic regions under selection for complex traits requires approaches more sensitive to detect subtle changes in allele frequencies over time and with greater flexibility to detect selection on segregating variants. At least in domestic animals, the explicit use of the pedigree structure in may be more appropriate to detect genomic regions responsible for recent selection e.g. [57, 58]. We did find a weak association between selection signatures (*|iHS|*) and QTL for milk production traits by considering 20% of the genome. However, finding such a weak association over such a large part of the genome is not very useful in practice. This weak association occurred despite the advantages of using genomic selection methodologies to identify QTL [11]. For example, compared to single SNP regressions, our approach to identify QTL can capture a higher proportion of the genetic variance [52] and has an improved ability to account for population stratification [59].

The detection of clear selection signatures is compromised by a number of other factors that are illustrated by the individual loci that we examined. There are many traits subject to natural and artificial selection and many genes affect each trait. Therefore the genome contains many possible sites of selection and this complicates the interpretation of the data. For instance, we examined the region surrounding *ABCG2* but may well have detected selection at *NCAPG-LCORL*. The large number of loci segregating for many traits possibly also leads to complex results on BTA20 where there are > 1 QTL for milk production [43]. Also multiple alleles at a locus under selection seems to be common and could cloud the interpretation. We found or confirmed multiple alleles at *POLLED*, *MC1-R*, *KIT*, *KITLG* and *PMEL*. Migration or introgression of a selected mutation from one breed to another leaves an unusual selection signature as shown by *PLAG1* in Limousin where *F*_*ST*_ between Limousin and other breeds is high except at the position of the selected mutation. This pattern is expected if the common ancestor of all *PLAG1* mutant alleles in Limousin is a Limousin haplotype that differs except at the *PLAG1* mutation from haplotypes in other breeds carrying the same mutation. In the case of *DGAT1* there has been recent selection for the ancestral allele after possible earlier selection for the mutant. Thus many of the small sample of genes studied display properties that complicate the interpretation of the data and decrease our ability to find clear evidence of classic selection signatures.

## Conclusions

We conclude that the conditions that give rise to a clear selection signatures (i.e. strong selection for a mutation that would previously have been detrimental) are rare. More usually the response to selection is based on small frequency changes at many loci that were already polymorphic in the population before selection began. Consequently, many of the claims for identifying loci affecting complex traits using selection signatures must be treated with caution.

## Methods

### Overview

We obtained real and imputed Illumina Bovine high-density genotypes from 8 cattle selected primarily for dairy or beef production (dairy breeds: Holstein, Jersey; Beef breeds: Angus, Charolais, Limousin, Hereford, Murray Grey, Shorthorn). Sliding windows of 250 kb were constructed across the genome, where each 250 kb length was separated by 50 kb. A window size of 250 kb was chosen because its approximate time to coalescence is 2,000 years (i.e. 1/0.0025 Morgan = 400 generations or 2,000 years assuming 5 years per generation; following [60]), which should represent chromosome segments segregating in domesticated cattle prior to breed formation. For each window, we calculated statistics which would identify within breed selection (i.e. *HAPH* and *|iHS|* defined below), computed the divergence between the breeds using Wright’s *F*_*ST*_ and calculated the variance in genomic estimated breeding values (GEBV) for Jersey and Holstein breeds for dairy traits (milk, fat and protein yield; fat and protein concentration; stature and fertility). We tested for over-representation of the top 5% of windows with selection signatures (within either Holstein or Jersey, and across dairy and beef breeds) that were also in the top 5% of windows for genetic variance in dairy traits. The significance of this over-representation was assessed by a chi-squared test on a 2x2 contingency table. The 3 selection statistics and annotated genomic features for each 250 kb window are contained in Additional file 1.

### Genotype data

Datasets from dairy and beef cattle were available for analysis. We analysed only autosomal SNP. The dairy dataset consisted of 616,350 SNP for 13,501 Holstein and 5240 Jersey animals. The beef dataset consisted of 692,527 SNP for 2510 Angus, 463 Charolais, 744 Hereford, 61 Limousin, 254 Murray Grey and 868 Shorthorn cattle. Genotype quality control and imputation methods for the dairy data are described by Erbe *et al.*[61] and Bolormaa *et al.*[62] describes the beef data.

### Within breed selection – haplotype homozygosity (*HAPH*)

Haplotype segments were constructed for dairy and beef datasets using phased data from Beagle [63] and non-overlapping segments of 30 or 31 SNP. For each chromosome segment we calculated a modified version of Depaulis-Veuille’s H-test [8], referred to as *HAPH*, where *HAPH* = , where *p*_*i*_ is the (within breed) frequency of the *i*^th^ haplotype and *N* is the total number of haplotypes observed for the breed at the position. Chromosome segments were allocated to 250 kb windows in which their mid-point fell and the average calculated for each 250 kb window. *HAPH* was then standardized by dividing this value by the breed average over all windows 'Hard sweeps' (i.e. Table 6) were identified by windows in the top 5% of *HAPH* values and separated by less than 1 Mb.

### Within breed selection – the integrated haplotype score (*|iHS|*)

*|iHS|* was calculated within breed for each SNP in dairy and beef datasets following Voight *et al.*[9]. *iHS* is a measure of haplotype homozygosity surrounding the derived allele at a SNP compared to the haplotype homozygosity surrounding the ancestral allele at the SNP. To determine the ancestral allele, genotypes for 750,948 SNP from the Bovine HD chip were obtained for 2 Banteng, 7 Bison and 8 Buffalo animals. All genotype calls were used and the ancestral allele was taken as the most frequent allele observed in these out-group animals. Only one allele was observed for most (85%) SNP. Next, the integrated extended haplotype homozygosity (*iEHH*) was calculated within breed for the ancestral and derived SNP allele using the ‘rehh’ package in R [64, 65]. The homozygosity decay threshold for *iEHH* was 0.5 and all SNP had a minor allele frequency > 0.001. Finally, the log_10_ ratio of *iEHH* for the ancestral compared to the derived allele was standardised to a mean of zero and standard deviation of 1 in 20 bins, where bins were determined by frequency of the ancestral allele [i.e. (*log*_10_*x* – *μ*)/*σ*, when *x* is the *iEHH* of the derived allele divided by the ancestral allele, and μ and σ are the mean and standard deviation of log_10_*iEHH* ratios for each bin]. The final statistic, the integrated haplotype score (*iHS*), therefore measured the haplotype homozygosity surrounding a derived SNP allele compared to that surrounding the ancestral SNP allele. Although a negative *iHS* indicates greater homozygosity surrounding the ancestral allele and a positive *iHS* indicates greater homozygosity surrounding the derived allele, we analysed the absolute value of *iHS* so that the measure was independent of the allele classification. This is because either SNP allele might be on the same chromosome segment as the causative mutation. The maximum value of *|iHS|* was used for each 250 kb window.

### Differentiation between breeds – calculation of *F*_*ST*_ for each breed by breed comparison

Wright’s measure of population differentiation (*F*_*ST*_) was calculated for each breed combination (i.e. 8 breeds = 28 comparisons) using a common set of 610,123 SNP. The average *F*_*ST*_ was calculated in each 250 kb window following Weir & Cockerham [66] as:1

where *j* is each SNP in the 250 kb window, *p*_*ij*_ is the allele frequency for breed *i* at SNP *j*, and  is the mean allele frequency of the breeds at SNP *j*. On average there were 60 SNP per window (range: 1 to 173 SNP; SD: 22 SNP).

To find windows where dairy breeds differed most from beef breeds the *F*_*ST*_ values between pairs of breeds where one was a dairy breed and one was a beef breed (e.g. Holstein with Angus) were compared to *F*_*ST*_ values between breeds where both were either dairy (Holstein with Jersey) or type 1 and type 2 loci beef breeds (e.g. Angus with Charolais). *F*_*ST*_ values for a window were divided by the mean *F*_*ST*_ over all windows for that pair of breeds and then compared using a one-sided non-parametric Mann–Whitney U test.

### Variance in GEBV for milk production traits

Phenotypes and genotypes were obtained from the Australian Dairy Herd Improvement Scheme (ADHIS) for 3,391 Holstein and 1,014 Jersey bulls. Bull genotypes were a subset of animals used to detect the selection signatures. The effect of each SNP was estimated using BayesR, using the same process as Erbe *et al.*[61], which simultaneously estimates the mean, a polygenic effect and the effects of all SNP. Separate analysis were conducted for each trait by breed combination, where each analysis used 50,000 iterations (30,000 discarded as burn in) and SNP effects were the mean of 5 replicate chains. For each trait we estimated the genetic value of each 250 kb window in each animal (its local GEBV) by  (i.e. *X* is a matrix of genotypes, and  is the estimated SNP effect from BayesR). The variance across animals of GEBVs at a window indicates the windows contribution to genetic variance for that trait. The windows with the top 5% of values for this variance for each breed by trait combination were assumed to contain putative QTL.

### Genomic annotations and selection of type 1 and type 2 loci

The locations of genomic features were downloaded using BioMart [67] on 15^th^ March 2013. Genes were mapped to each 250-kb window using their gene start and stop positions using their Ensemble ID and associated gene name (when available). All map positions of SNP and genomic features used UMD3. The loci used as type 1 and type 2 loci were a selection of loci available from the literature, including some identified from the Online Inheritance in Animals [10] database.

### Testing for over-representation of selection signatures with QTL for production traits

The top 5% of windows for *HAPH*, *|iHS|* and the dairy by beef *F*_*ST*_ test were deemed to indicate evidence of selection. A chi-squared test with 1 df was used to determine if the number of windows which ranked in the top 5% for the indicator of selection and the top 5% for the variance in GEBV for the production trait was more than expected by chance. The chi-squared test used the average of 5 non-overlapping sets of windows by dividing the actual number of overlapping windows by 5 (i.e. the number of times each segment of the genome was counted in a window). For the dairy by beef breed comparison, windows were counted if they were in the top 5% of windows for GEBV variance in either Holstein or Jersey.

### Ethics statement

No animal experiments were performed specifically for this manuscript. Where data were obtained from existing sources, references for these experiments are provided.

## Electronic supplementary material

Additional file 1: **This file contains the estimated haplotype homozygosity (** ***HAPH*** **)** ***,*** **the integrated haplotype score (** ***|iHS|*** **) and pairwise breed comparisons for Wright’s measure of population differentiation (** ***F***_***ST***_ **) at all 250 kb windows.** The data columns are defined in Additional file 3. The file is compressed with gzip. (ZIP 16 MB)

Additional file 2: **This file contains the column headers for** **Additional file** **1** **.** (TXT 1 KB)

Additional file 3: **This file contains** **Figures S1-S14** **and** **Tables S1-S3.** (DOCX 4 MB)
