# Supplementary material for: Selection for complex traits leaves little or no classic signatures of selection
Source: BMC Genomics. 2014 Mar 28;15(1):246. doi: 10.1186/1471-2164-15-246 (PMC3986643; doi:10.1186/1471-2164-15-246)
Supplement: Supplementary file 3 — Additional file 3: This file contains Figures S1-S14 and Tables S1-S3. (DOCX 4 MB) [file 12864_2014_5904_MOESM3_ESM.docx]

Additional file 2

Selection for complex traits leaves little or no classic signatures of selection

Kathryn E Kemper^1§^, Sarah J Saxton ^1,2^, Sunduimijid Bolormaa^3^, Benjamin J Hayes^3,4,5^, Michael E Goddard^1,3^

^1^Department of Agriculture and Food Systems, University of Melbourne, Parkville 3010, Australia

^2^Australian Dairy Herd Improvement Scheme, 22 William Street, Melbourne 3000, Australia

^3^Department of Environment and Primary Industries, AgriBio, Bundoora 3068, Australia

^4^La Trobe University, Bundoora 3086, Australia

^5^Dairy Futures Co-operative Research Centre, Bundoora 3068, Australia

^§^Corresponding author

Email addresses:

KEK: [kathryn.kemper@depi.vic.gov.au](mailto:kathryn.kemper@depi.vic.gov.au)

SJS: [ssaxton@adhis.com.au](mailto:ssaxton@adhis.com.au)

SB: [bolormaa.sunduimijid@depi.vic.gov.au](mailto:bolormaa.sunduimijid@depi.vic.gov.au)

BJH: [ben.hayes@depi.vic.gov.au](mailto:ben.hayes@depi.vic.gov.au)

MEG: [mike.goddard@depi.vic.gov.au](mailto:mike.goddard@depi.vic.gov.au)

| A. |  |
| --- | --- |
| B. |  |
| C. |  |

## Figure S1 - Genetic trends for milk (A), fat (B) and protein (C) yield in Holstein and Jersey cattle recorded in the Australian Dairy Herd Improvement scheme.

Note the different base populations for Holstein and Jersey cattle, hence direct comparisons between the breeds does not indicate phenotypic differences.

| A. |  |
| --- | --- |
| B. |  |

## Figure S2 - Genetic trends for fat (A) and protein (B) percentage in milk for Holstein and Jersey cattle recorded in the Australian Dairy Herd Improvement scheme.

Note the different base populations for Holstein and Jersey cattle, hence direct comparisons between the breeds does not indicate phenotypic differences.

| A. |  |
| --- | --- |
| B. |  |

## Figure S3 - Genetic trends for fertility (A) and stature (B) for Holstein and Jersey cattle recorded in the Australian Dairy Herd Improvement scheme.

Note the different base populations for Holstein and Jersey cattle, hence direct comparisons between the breeds does not indicate phenotypic differences.


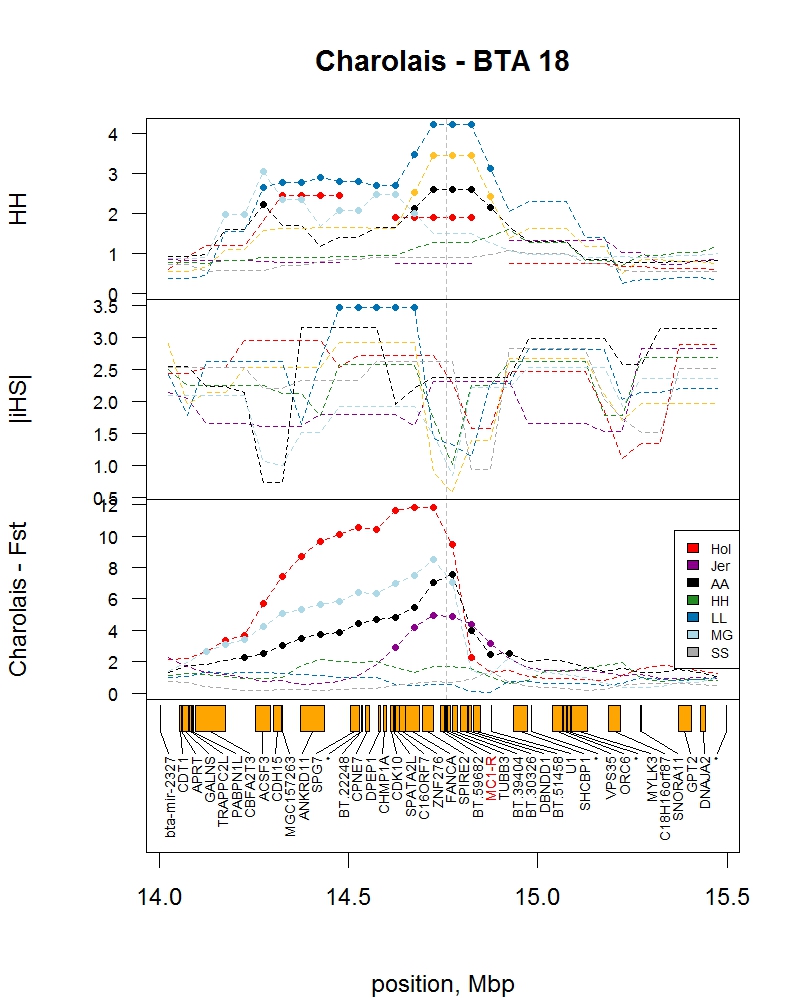


## Figure S4 - Haplotype homozygosity (*HAPH*) and integrated haplotype score *|iHS|* for all breeds, with *F_ST_* for Charolais contrasts at *MC1-R*.

Although several breeds show within breed selection, *F_ST_* indicates haplotype groups for 1. Holstein (Hol), Angus (AA), and Murray Grey (MG) [likely *E^D^* allele carriers]; 2. Hereford (HH), Limousin (LL) and Shorthorn (SS) [similar to Charolais and likely *e* allele breeds] and 3. Jersey [wild-type allele]. Points indicate windows with extreme *HAPH*, *|iHS|* or *F_ST_* (top 5% of windows).


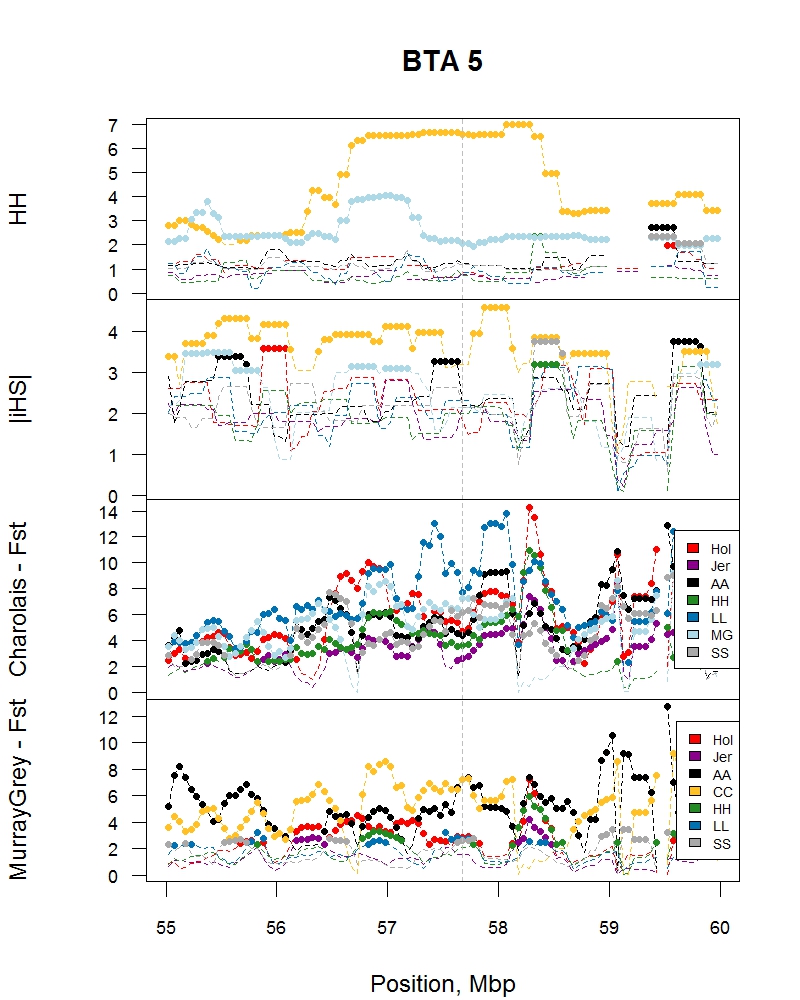


## Figure S5 - Haplotype homozygosity (*HAPH*) and integrated haplotype score (*|iHS|*) for all breeds near *PMEL* (57.67 Mbp, as indicated by dashed line), with *F_ST_* for Charolais and Murray Grey contrasts.

Both Charolais and Murray Grey indicate selection with *HAPH* (top 5% of windows, highlighted by points), but show differentiation via *F_ST_* near *PMEL*. Breeds are Hol = Holstein, Jer = Jersey, AA = Angus, CC = Charolais, HH = Hereford, LL = Limousin, MG = Murray Grey, SS = Shorthorn.


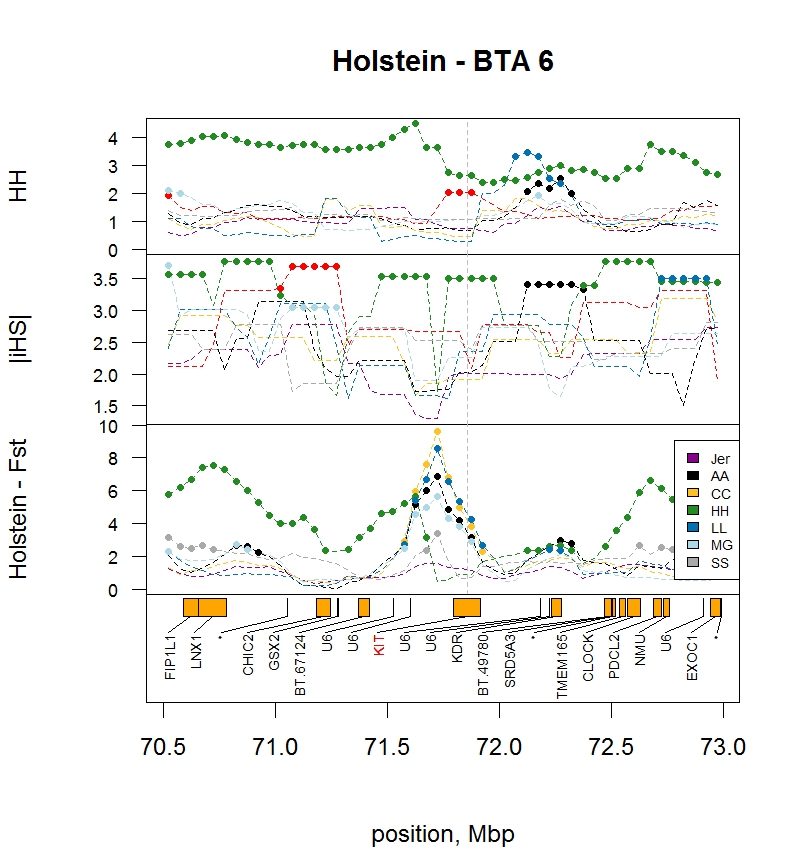


## Figure S6 - Haplotype homozygosity (*HAPH*) and integrated haplotype score (*|iHS|*) for all breeds, with *F_ST_* contrasts with Holstein near *KIT*.

Both Hereford and Holstein indicate within breed selection (top 5% of windows, highlighted by points) but for different haplotypes near *KIT*, particularly in the promoter region. Breeds are Jer = Jersey, AA = Angus, CC = Charolais, HH = Hereford, LL = Limousin, MG = Murray Grey, SS = Shorthorn.

##
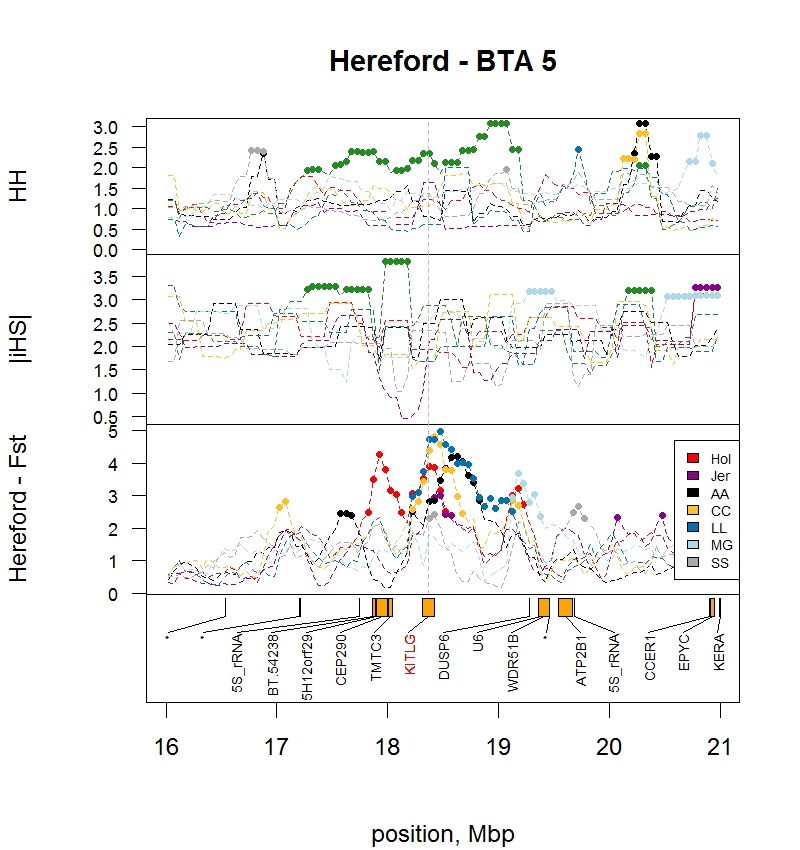
Figure S7 - Haplotype homozygosity (*HAPH*) and integrated haplotype score (*|iHS|*) for all breeds, with *F_ST_* contrasts with Hereford near *KITLG*.

Hereford indicates within breed selection with *HAPH* (top 5% of windows, highlighted by points) and differentiation with most other breeds via *F_ST_*. Breeds are Hol = Holstein, Jer = Jersey, AA = Angus, CC = Charolais, LL = Limousin, MG = Murray Grey, SS = Shorthorn.

##
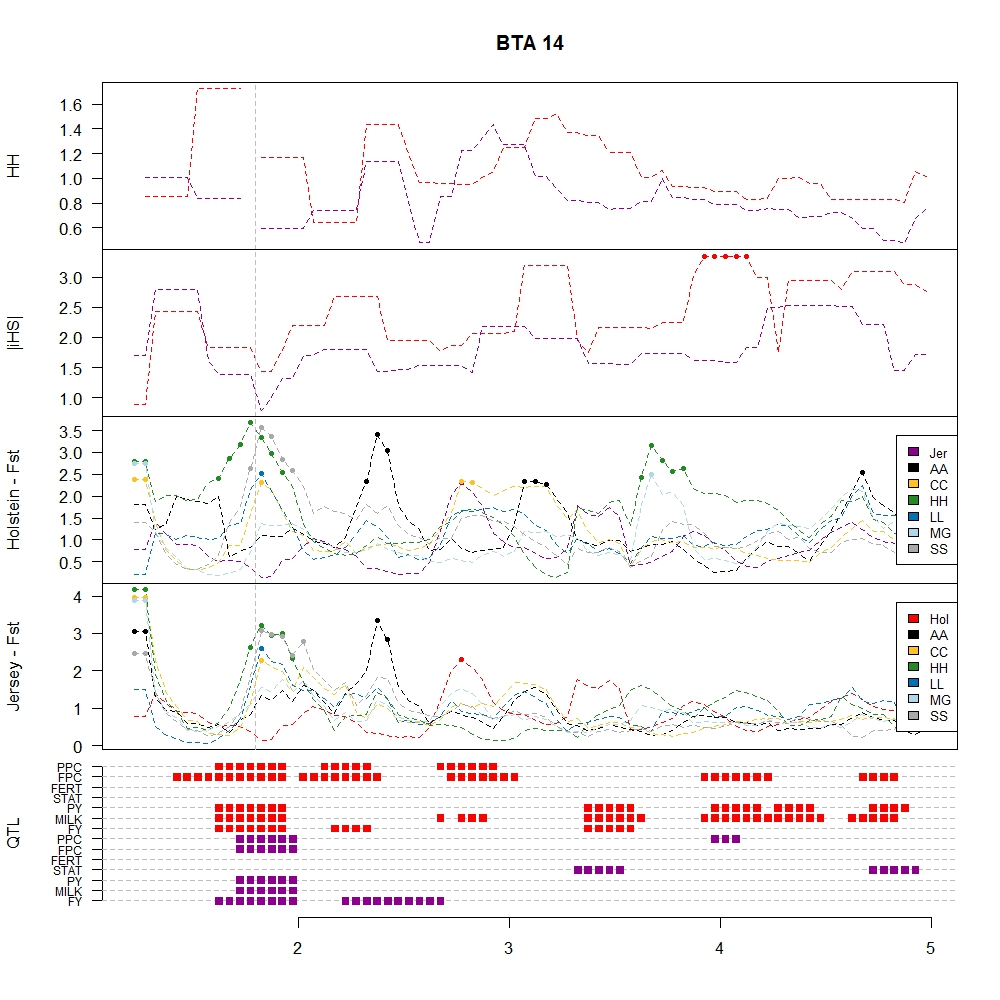
Figure S8 - Haplotype homozygosity (*HAPH*), integrated haplotype score (*|iHS|*), *F_ST_* with Jersey or Holstein and production trait quantitative trait loci (QTL) for Jersey or Holstein near *DGAT1* (1.80 Mbp, as indicated by the dashed vertical line).

Breeds are Hol = Holstein, Jer = Jersey, AA = Angus, CC = Charolais, HH = Hereford, LL = Limousin, MG = Murray Grey, SS = Shorthorn. QTL traits are for fat (FY), milk (MILK) and protein (PY) yield, stature (STAT), fertility (FERT), and fat (FPC) and protein (PPC) percentage in milk for Jersey (purple) and Holstein (red). Windows with extreme values (in the top 5% per trait or statistic) are highlighted with points.

##
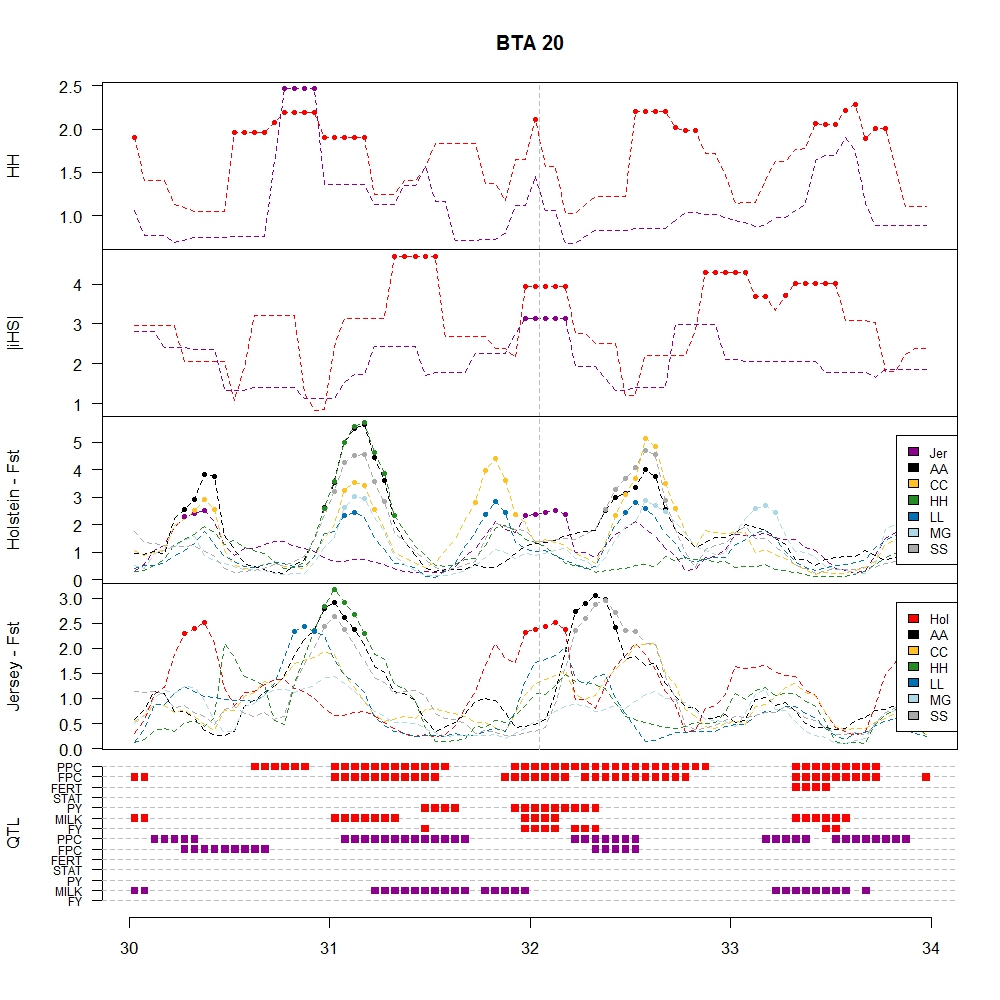
Figure S9 - Haplotype homozygosity (*HAPH*), integrated haplotype score (*|iHS|*), *F_ST_* with Jersey or Holstein and production trait quantitative trait loci (QTL) for Jersey or Holstein near the *GHR* locus (32.05 Mbp, as indicated by the vertical dashed line).

Breeds are Hol = Holstein, Jer = Jersey, AA = Angus, CC = Charolais, HH = Hereford, LL = Limousin, MG = Murray Grey, SS = Shorthorn. QTL traits are for fat (FY), milk (MILK) and protein (PY) yield, stature (STAT), fertility (FERT), and fat (FPC) and protein (PPC) percentage in milk for Jersey (purple) and Holstein (red) cattle. Windows with extreme values (in the top 5% per trait or statistic) are highlighted with points.

##
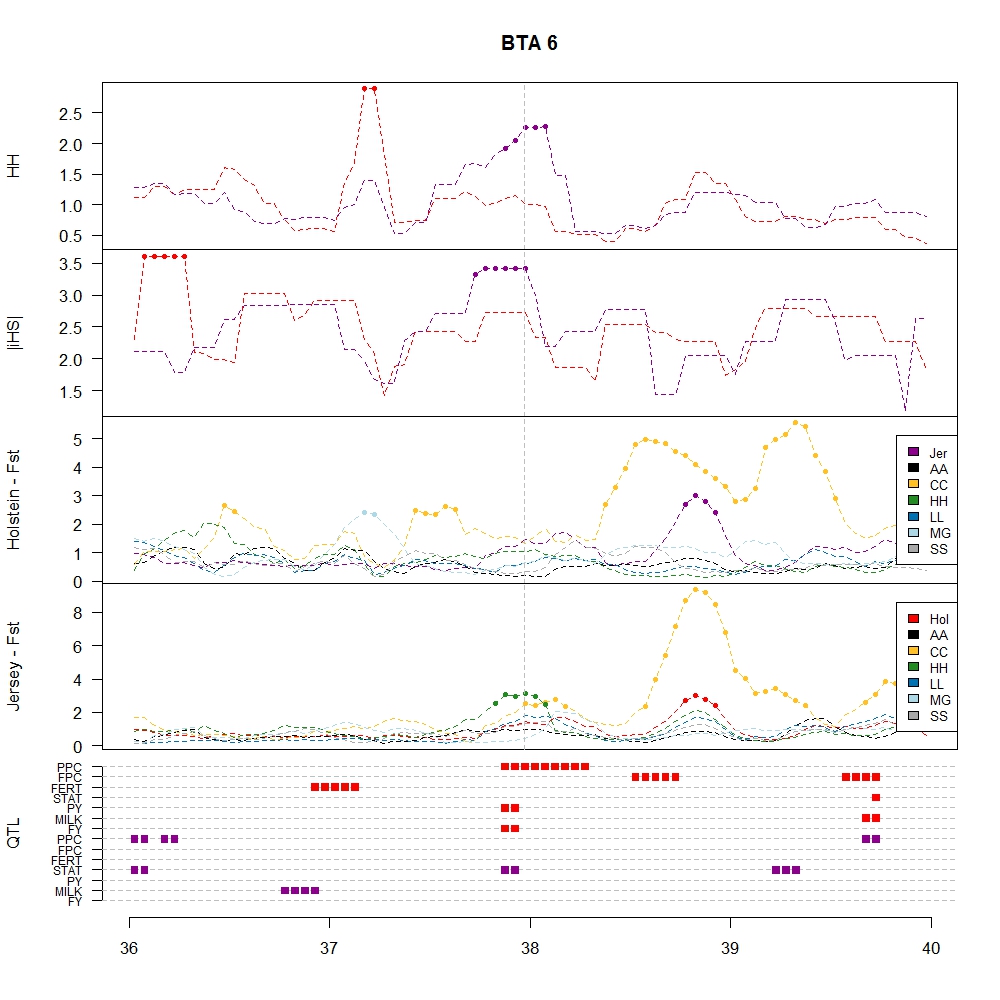
Figure S10 - Haplotype homozygosity (*HAPH*), integrated haplotype score (*|iHS|*), *F_ST_* with Jersey or Holstein and production trait quantitative trait loci (QTL) for Jersey or Holstein near *ABCG2* (37.97 Mbp).

Breeds are Hol = Holstein, Jer = Jersey, AA = Angus, CC = Charolais, HH = Hereford, LL = Limousin, MG = Murray Grey, SS = Shorthorn. QTL traits are for fat (FY), milk (MILK) and protein (PY) yield, stature (STAT), fertility (FERT), and fat (FPC) and protein (PPC) percentage in milk for Jersey (purple) and Holstein (red) cattle. Windows with extreme values (in the top 5% per trait or statistic) are highlighted with points. Note that *NCAPG* (Non-SMC condensin I complex, subunit G) is at 38.78 Mbp and is probably responsible for the differentiation in Charolais.


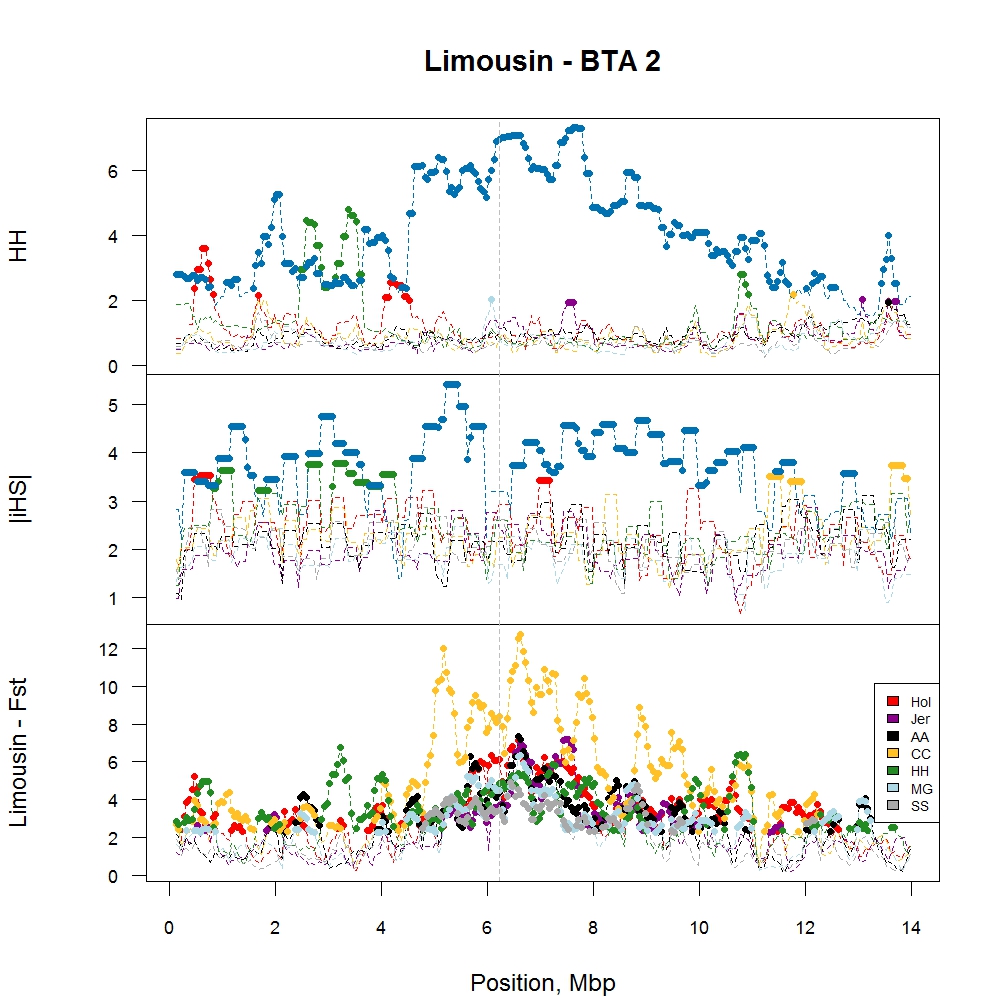


## Figure S11 - Haplotype homozygosity (*HAPH*) and integrated haplotype score (*|iHS|*) for all breeds with *F_ST_* contrasts with Limousin.

Windows with extreme values (in the top 5%) are highlighted with points. Breeds are Hol = Holstein, Jer = Jersey, AA = Angus, CC = Charolais, HH = Hereford, LL = Limousin, MG = Murray Grey, SS = Shorthorn.


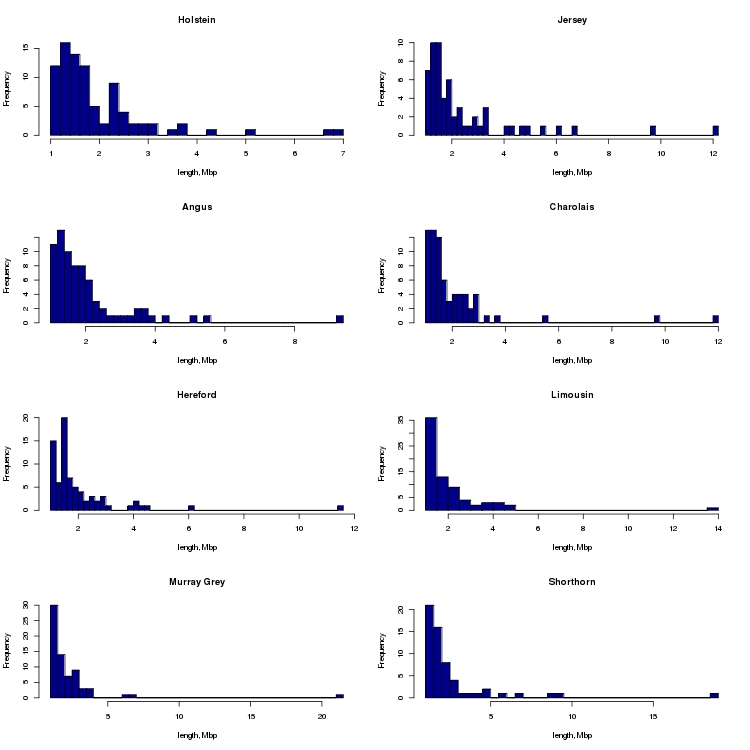


## Figure S12 - Distribution of hard sweep lengths (> 1 Mbp) identified as with extreme (top 5%) haplotype homozygosity (*HAPH*) for Holstein, Jersey, Angus, Charolais, Hereford, Limousin, Murray Grey and Shorthorn breeds (L-R, top-bottom).

##
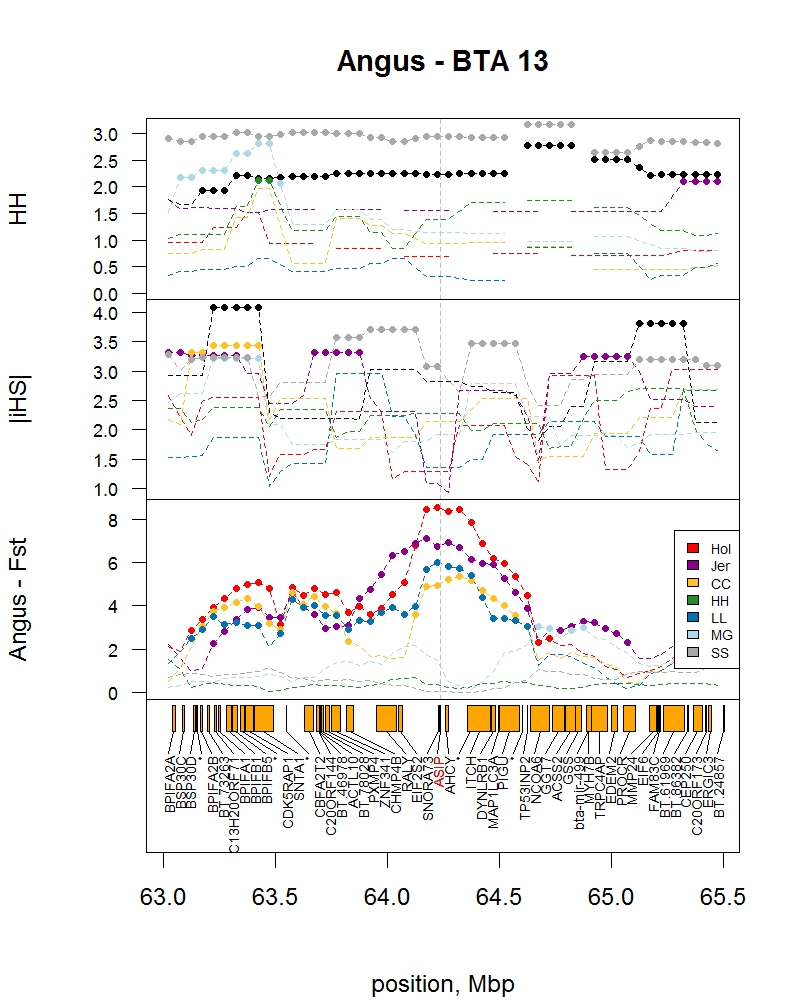
Figure S13 - Haplotype homozygosity (*HAPH*) and integrated haplotype score (*|iHS|*) for all breeds with *F_ST_* contrasts with Angus.

Windows with extreme values (in the top 5%) are highlighted with points. Breeds are Hol = Holstein, Jer = Jersey, AA = Angus, CC = Charolais, HH = Hereford, LL = Limousin, MG = Murray Grey, SS = Shorthorn.

##
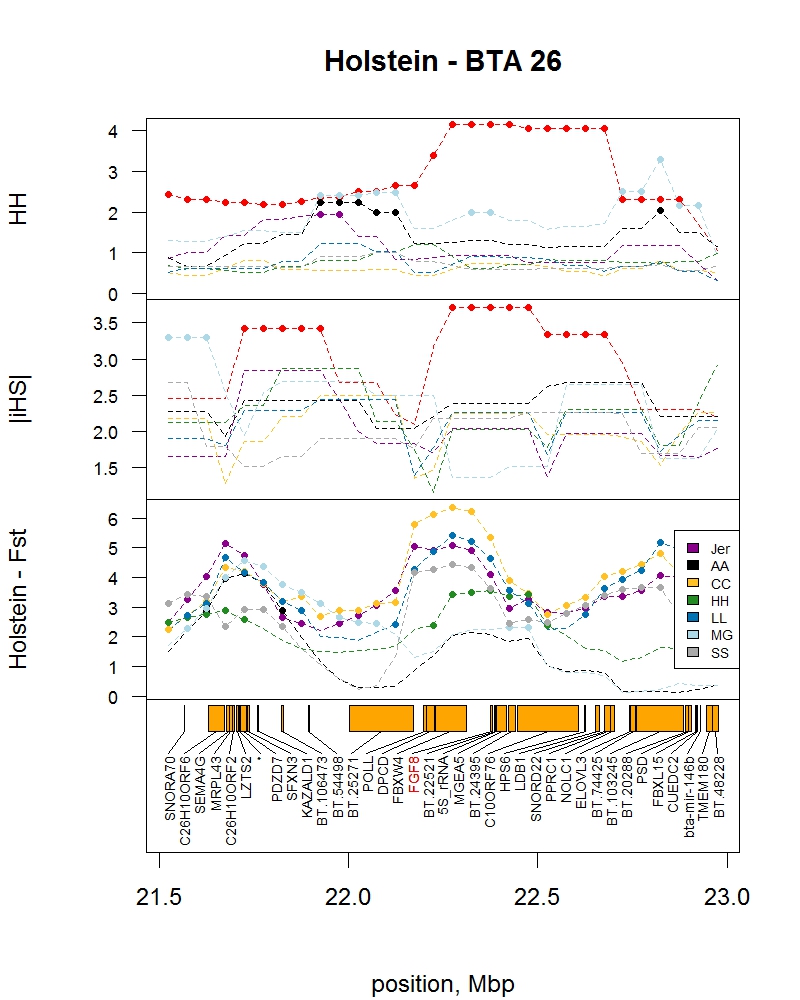
Figure S14 - Haplotype homozygosity (*HAPH*) and integrated haplotype score (*|iHS|*) for all breeds with *F_ST_* contrasts with Holstein.

Windows with extreme values (in the top 5%) are highlighted with points. Breeds are Hol = Holstein, Jer = Jersey, AA = Angus, CC = Charolais, HH = Hereford, LL = Limousin, MG = Murray Grey, SS = Shorthorn.

## Table S1 - Chi-squared tests for over-representation of measures of selection (haplotype homozygosity, *HAPH*; integrated haplotype score, *|iHS|*) with Holstein QTL for fat, milk and protein yield (FY, MILK, PY), stature (STAT), fertility (FERT) and precent of fat and protein in milk (FPC, PPC). The final row tests the overlap between the two measures.

| Grp1 | Grp2 | Observed | | | | Expected | | | | $\sum\frac{\left( obs-Exp \right)^{2}}{Exp}$ | P-val | Bonf. Corr. |
| --- | --- | --- | --- | --- | --- | --- | --- | --- | --- | --- | --- | --- |
|  |  | Gp1+Gp2 | Gp1 | Gp2 | Total | Gp1+G2 | Gp1 | Gp2 | Total |  |  |  |
| H.FY | H.HAPH | 31.4 | 499.2 | 633.4 | 9871.8 | 32.0 | 601.4 | 467.2 | 8771.2 | 0.014 | 0.9060 |  |
| H.FY | H.iHS | 40.0 | 499.8 | 526.2 | 9994.0 | 26.3 | 499.9 | 473.5 | 8994.3 | 7.907 | 0.0049 |  |
| H.MILK | H.HAPH | 32.8 | 499.6 | 633.4 | 9871.8 | 32.1 | 601.3 | 467.5 | 8770.9 | 0.019 | 0.8891 |  |
| H.MILK | H.iHS | 39.0 | 499.8 | 526.2 | 9994.0 | 26.3 | 499.9 | 473.5 | 8994.3 | 6.794 | 0.0091 |  |
| H.PY | H.HAPH | 35.6 | 499.2 | 633.4 | 9871.8 | 32.0 | 601.4 | 467.2 | 8771.2 | 0.448 | 0.5034 |  |
| H.PY | H.iHS | 47.0 | 499.8 | 526.2 | 9994.0 | 26.3 | 499.9 | 473.5 | 8994.3 | 18.066 | 0.0000 | Sig. |
| H.STAT | H.HAPH | 34.0 | 498.0 | 633.4 | 9871.8 | 32.0 | 601.4 | 466.0 | 8772.4 | 0.148 | 0.7009 |  |
| H.STAT | H.iHS | 40.2 | 499.8 | 526.2 | 9994.0 | 26.3 | 499.9 | 473.5 | 8994.3 | 8.140 | 0.0043 |  |
| H.FERT | H.HAPH | 31.0 | 499.4 | 633.4 | 9871.8 | 32.0 | 601.4 | 467.4 | 8771.0 | 0.038 | 0.8451 |  |
| H.FERT | H.iHS | 39.6 | 499.8 | 526.2 | 9994.0 | 26.3 | 499.9 | 473.5 | 8994.3 | 7.452 | 0.0063 |  |
| H.FPC | H.HAPH | 30.6 | 498.6 | 633.4 | 9871.8 | 32.0 | 601.4 | 466.6 | 8771.8 | 0.068 | 0.7941 |  |
| H.FPC | H.iHS | 36.0 | 499.8 | 526.2 | 9994.0 | 26.3 | 499.9 | 473.5 | 8994.3 | 3.960 | 0.0466 |  |
| H.PPC | H.HAPH | 30.8 | 497.6 | 633.4 | 9871.8 | 31.9 | 601.5 | 465.7 | 8772.7 | 0.045 | 0.8324 |  |
| H.PPC | H.iHS | 34.6 | 499.8 | 526.2 | 9994.0 | 26.3 | 499.9 | 473.5 | 8994.3 | 2.898 | 0.0887 |  |
| H.HH | H.iHS | 75.6 | 494.0 | 526.2 | 9871.8 | 26.3 | 499.9 | 467.7 | 8877.9 | 102.503 | 0.0000 | Sig. |

## Table S2 - Chi-squared tests for over-representation of measures of selection (haplotype homozygosity, *HAPH*; integrated haplotype score, *|iHS|*) with Jersey QTL for fat, milk and protein yield (FY, MILK, PY), stature (STAT), fertility (FERT) and precent of fat and protein in milk (FPC, PPC). The final row tests the overlap between the two measures.

| Grp1 | Grp2 | Observed | | | | Expected | | | | $\sum\frac{\left( obs-Exp \right)^{2}}{Exp}$ | P-val | Bonf. Corr. |
| --- | --- | --- | --- | --- | --- | --- | --- | --- | --- | --- | --- | --- |
|  |  | Gp1+Gp2 | Gp1 | Gp2 | Total | Gp1+G2 | Gp1 | Gp2 | Total |  |  |  |
| J.FY | J.HAPH | 21.4 | 499.6 | 633.6 | 9871.8 | 32.1 | 601.5 | 467.5 | 8770.7 | 3.993 | 0.0457 |  |
| J.FY | J.iHS | 31.8 | 499.8 | 526.6 | 9994.0 | 26.3 | 500.3 | 473.5 | 8993.9 | 1.260 | 0.2616 |  |
| J.MILK | J.HAPH | 25.2 | 499.6 | 633.6 | 9871.8 | 32.1 | 601.5 | 467.5 | 8770.7 | 1.655 | 0.1983 |  |
| J.MILK | J.iHS | 36.4 | 499.8 | 526.6 | 9994.0 | 26.3 | 500.3 | 473.5 | 8993.9 | 4.274 | 0.0387 |  |
| J.PY | J.HAPH | 29.2 | 499.2 | 633.6 | 9871.8 | 32.0 | 601.6 | 467.2 | 8771.0 | 0.283 | 0.5945 |  |
| J.PY | J.iHS | 35.0 | 499.8 | 526.6 | 9994.0 | 26.3 | 500.3 | 473.5 | 8993.9 | 3.168 | 0.0751 |  |
| J.STAT | J.HAPH | 22.6 | 499.8 | 633.6 | 9871.8 | 32.1 | 601.5 | 467.7 | 8770.5 | 3.152 | 0.0758 |  |
| J.STAT | J.iHS | 43.0 | 499.8 | 526.6 | 9994.0 | 26.3 | 500.3 | 473.5 | 8993.9 | 11.718 | 0.0006 | Sig. |
| J.FERT | J.HAPH | 20.4 | 499.6 | 633.6 | 9871.8 | 32.1 | 601.5 | 467.5 | 8770.7 | 4.777 | 0.0288 |  |
| J.FERT | J.iHS | 34.2 | 499.8 | 526.6 | 9994.0 | 26.3 | 500.3 | 473.5 | 8993.9 | 2.610 | 0.1062 |  |
| J.FPC | J.HAPH | 16.6 | 498.8 | 633.6 | 9871.8 | 32.0 | 601.6 | 466.8 | 8771.4 | 8.353 | 0.0039 |  |
| J.FPC | J.iHS | 28.6 | 499.8 | 526.6 | 9994.0 | 26.3 | 500.3 | 473.5 | 8993.9 | 0.216 | 0.6418 |  |
| J.PPC | J.HAPH | 19.8 | 498.8 | 633.6 | 9871.8 | 32.0 | 601.6 | 466.8 | 8771.4 | 5.245 | 0.0220 |  |
| J.PPC | J.iHS | 27.6 | 499.8 | 526.6 | 9994.0 | 26.3 | 500.3 | 473.5 | 8993.9 | 0.067 | 0.7950 |  |
| J.HH | J.iHS | 130.4 | 494.2 | 526.6 | 9871.8 | 26.4 | 500.2 | 467.8 | 8877.4 | 456.567 | 0.0000 | Sig. |

## Table S3 - Chi-squared tests for over-representation of selection between dairy and beef breeds (*F_ST_*) with Holstein or Jersey QTL for fat, milk and protein yield (FY, MILK, PY), stature (STAT), fertility (FERT) and precent of fat and protein in milk (FPC, PPC). The final rows test the overlap between the QTL in Holstein and Jersey.

| Grp1 | Grp2 | Observed | | | | Expected | | | | $\sum\frac{\left( obs-Exp \right)^{2}}{Exp}$ | P-val | Bonf. Corr. |
| --- | --- | --- | --- | --- | --- | --- | --- | --- | --- | --- | --- | --- |
|  |  | Gp1+Gp2 | Gp1 | Gp2 | Total | Gp1+G2 | Gp1 | Gp2 | Total |  |  |  |
| H/J.FY | Fst | 55.2 | 943.4 | 472.8 | 9979.4 | 44.7 | 428.1 | 898.7 | 8607.9 | 2.862 | 0.0907 |  |
| H/J.MILK | Fst | 47.0 | 958.6 | 472.8 | 9979.4 | 45.4 | 427.4 | 913.2 | 8593.4 | 0.064 | 0.8001 |  |
| H/J.PY | Fst | 48.0 | 956.2 | 472.8 | 9979.4 | 45.3 | 427.5 | 910.9 | 8595.7 | 0.186 | 0.6659 |  |
| H/J.STAT | Fst | 42.6 | 947.4 | 472.8 | 9979.4 | 44.9 | 427.9 | 902.5 | 8604.1 | 0.135 | 0.7133 |  |
| H/J.FERT | Fst | 44.0 | 949.0 | 472.8 | 9979.4 | 45.0 | 427.8 | 904.0 | 8602.6 | 0.024 | 0.8773 |  |
| H/J.FPC | Fst | 44.0 | 956.8 | 472.8 | 9979.4 | 45.3 | 427.5 | 911.5 | 8595.1 | 0.045 | 0.8313 |  |
| H/J.PPC | Fst | 45.8 | 967.2 | 472.8 | 9979.4 | 45.8 | 427.0 | 921.4 | 8585.2 | 0.000 | 0.9970 |  |
| H.FY | J.FY | 46.0 | 499.8 | 527.0 | 9994.0 | 26.4 | 500.6 | 473.4 | 8993.6 | 16.272 | 0.0001 | Sig. |
| H.MILK | J.MILK | 47.6 | 499.8 | 527.0 | 9994.0 | 26.4 | 500.6 | 473.4 | 8993.6 | 19.030 | 0.0000 | Sig. |
| H.PY | J.PY | 47.6 | 499.8 | 527.0 | 9994.0 | 26.4 | 500.6 | 473.4 | 8993.6 | 19.030 | 0.0000 | Sig. |
| H.STAT | J.STAT | 51.6 | 499.8 | 527.0 | 9994.0 | 26.4 | 500.6 | 473.4 | 8993.6 | 26.871 | 0.0000 | Sig. |
| H.FERT | J.FERT | 34.2 | 499.8 | 527.0 | 9994.0 | 26.4 | 500.6 | 473.4 | 8993.6 | 2.595 | 0.1072 |  |
| H.FPC | J.FPC | 50.4 | 499.8 | 527.0 | 9994.0 | 26.4 | 500.6 | 473.4 | 8993.6 | 24.377 | 0.0000 | Sig. |
| H.PPC | J.PPC | 55.2 | 499.8 | 527.0 | 9994.0 | 26.4 | 500.6 | 473.4 | 8993.6 | 35.081 | 0.0000 | Sig. |
